# Smartphone-Based Assessment of Postural Balance in Patients With End-Stage Knee Osteoarthritis: A Cross-Sectional Study

**DOI:** 10.7759/cureus.86067

**Published:** 2025-06-15

**Authors:** José Albarello, Gustavo Halmenschlager, Yan Razuck, Conrado T Laett, Sidnei C da Silva, Thiago Lemos

**Affiliations:** 1 Laboratory of Neuromuscular Research and Exercise Physiology, National Institute of Traumatology and Orthopedics, Rio de Janeiro, BRA

**Keywords:** accelerometry, falls, physical function, postural control, wearable technology

## Abstract

Background

Osteoarthritis is the most prevalent joint disease and significantly impacts quality of life, particularly by increasing the risk of falls due to balance impairments. Individuals with knee osteoarthritis often experience musculoskeletal deficits that compromise proprioception and postural stability. While traditional tools for assessing balance, such as force platforms and motion capture systems, are effective, they are also costly and less accessible in clinical settings. Advances in mobile technology have enabled the use of smartphone-based inertial sensors as a practical alternative for evaluating postural control. However, evidence supporting their application in patients with end-stage knee osteoarthritis remains limited, especially regarding their association with physical function.

Materials and methods

This cross-sectional study included 40 participants, divided into knee osteoarthritis (n = 20) and control (n = 20) groups. Participants completed four static balance tasks while acceleration data were recorded using a smartphone. The tasks consisted of semi-tandem and parallel feet stances, performed with eyes open and closed. Each task was repeated twice for 30 seconds, with a one-minute rest between trials. In the knee osteoarthritis group, physical function was further assessed using the Timed Up and Go (TUG) test and the 30-second chair stand test. Acceleration data were processed to compute the root mean square values for body sway in the antero-posterior and medio-lateral directions, as well as overall acceleration magnitude.

Results

The Mann-Whitney U-test revealed significant differences between groups in most tasks and parameters, with the knee osteoarthritis group exhibiting significantly higher body acceleration. Furthermore, increased body sway was significantly correlated with poorer physical function, particularly in semi-tandem tasks.

Conclusion

Our findings suggest that smartphone-derived measurements provide an effective means of assessing postural control in patients with end-stage knee osteoarthritis, offering valuable insights for managing fall risk and informing tailored rehabilitation strategies.

## Introduction

Osteoarthritis, the most prevalent joint disease globally [1], is primarily driven by factors such as obesity and aging [2], significantly impacting quality of life by increasing years lived with disability. Patients with osteoarthritis are often at a heightened risk of falls compared to their healthy counterparts, due to musculoskeletal impairments that adversely affect proprioception and balance [3]. Previous evidence indicates that between 24% and 48% of patients with end-stage knee osteoarthritis awaiting total knee arthroplasty have experienced falls [4,5]. Therefore, addressing postural balance in knee osteoarthritis patients is crucial for managing fall risk and preventing further complications.

The assessment of balance aims to evaluate and monitor the progression of postural control, providing valuable insights that allow therapists to tailor interventions and manage fall risk more effectively [6]. However, gold-standard methods for assessing postural control, such as force platforms [7] and motion capture systems [8], are costly and require specialized scientific expertise to interpret results. While these instruments are widely utilized in research, they are generally inaccessible to many therapists in clinical settings. In practice, balance performance is commonly assessed through either the maximum time an individual can sustain a specific task [9], or score-based instruments like the Berg Balance Scale [10]. Although these clinical outcomes are quick, user-friendly, and practical, their scoring criteria are often subjective and fail to capture postural adjustments throughout the task. To overcome these limitations and offer more accessible, quantitative assessments, technological advancements in inertial sensors have enabled the use of smartphones to monitor postural control characteristics [6].

Smartphones are widely available, portable, and cost-effective, making them ideal for balance assessment in clinical settings. The triaxial accelerometer embedded in smartphones has been validated for static balance assessment in healthy young adults, showing a strong positive correlation with force platform measurements [11]. Additionally, smartphone-based assessments have demonstrated excellent between-days reliability in static balance evaluations and effectively distinguish between tasks of varying difficulty levels, such as eyes open/closed and unipedal/bipedal support [12]. This technology has also been applied to various clinical populations, including individuals with Parkinson's disease [13], multiple sclerosis [14], and the elderly population [15]. Despite the growing research interest over the last decade, studies employing smartphone-based inertial sensors to assess balance in patients with knee osteoarthritis are still lacking. Moreover, it remains unclear whether measurements of body sway derived from smartphones correlate with the performance of functional tests in this patient population.

In this study, we aim to evaluate the ability of smartphone-derived measurements to differentiate patients with end-stage knee osteoarthritis from healthy controls and to assess their relationship with functional test performance. Specifically, acceleration data were acquired using a smartphone during four distinct static balance tasks, and body sway variables were compared between the groups. Additionally, we analyzed the correlation between body sway variables and performance on the Timed Up and Go (TUG) test and the 30-second chair stand test in the knee osteoarthritis group. Given the propensity for osteoarthritis to impair balance, we hypothesized that individuals with knee osteoarthritis would exhibit increased body sway relative to the control group, and that these sway measurements would correlate with their performance on functional tests.

## Materials and methods

Study design

This cross-sectional study involved a single 70-minute laboratory visit. Participants with knee osteoarthritis were recruited during their hospitalization, one day before undergoing total knee arthroplasty. Initially, participants provided informed consent after reading the study information. They were then familiarized with the postural balance tasks until they felt comfortable performing them. Following these tasks, the TUG test and the 30-second chair stand test were thoroughly explained to each participant, as only one trial for each test was conducted. A three-minute rest period was provided between the functional tests to ensure adequate recovery. All experimental procedures adhered to the Declaration of Helsinki and received approval from the local Ethics Committee (approval number: 6.686.691; approval date: March 5, 2024).

Participants

A priori sample size calculation was conducted using G*Power (version 3.1.9.7; Heinrich-Heine-Universität Düsseldorf, Düsseldorf, Germany) for a correlation analysis based on a bivariate normal model. With a significance level of 0.05 and a correlation coefficient of 0.5, the analysis indicated that a minimum sample size of 18 patients with knee osteoarthritis would be necessary to achieve a statistical power of 70%. Thus, 40 participants were recruited and divided into two groups: the knee osteoarthritis group and the control group.

The knee osteoarthritis group consisted of 20 individuals (12 females), with a mean ± standard deviation (SD) age of 61 ± 7 years, body mass of 78.56 ± 17.71 kg, and height of 162.95 ± 9.59 cm. Participants in this group were diagnosed with unilateral end-stage knee osteoarthritis, classified according to the modified Ahlbäck classification [16]. Patients were eligible if scheduled for total knee arthroplasty and excluded if they presented neurological diseases, vestibular disorders, cognitive impairments preventing test comprehension, previous surgery on the affected knee, or musculoskeletal injuries affecting the lower limbs in the last six months. The control group included 20 physically active participants (nine females), with a mean ± SD age of 31 ± 10 years, body mass of 74.20 ± 18.87 kg, and height of 170.50 ± 10.48 cm. Individuals were excluded from the control group if they reported lower limb musculoskeletal injuries in the preceding six months or exhibited impairments affecting postural control. The study was conducted throughout the year 2024.

Postural balance tasks

**Figure 1:**
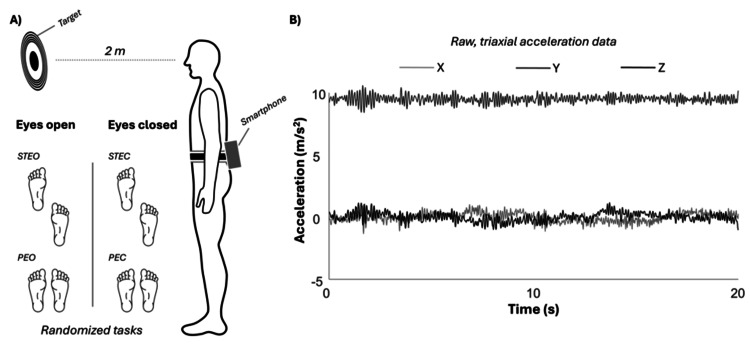
Experimental procedures and acceleration signal acquisition (A) Participants performed four postural balance tasks following a random order: STEO and STEC; and PEO and PEC. For the tasks performed with eyes open, participants were instructed to fix their gaze on a target at eye level, positioned two meters away (upper part). (B) The 3D acceleration signal was acquired from a smartphone during the postural balance tasks. Image credit for Figure (A): José Albarello Sr. STEO, semi-tandem with eyes open; STEC, semi-tandem with eyes closed; PEO, parallel feet with eyes open; PEC, parallel feet with eyes closed

A smartphone was positioned on the lower back and fixed using an elastic belt. Four tasks were performed in random order to assess static balance: semi-tandem with (1) eyes open and (2) eyes closed; and parallel feet with (3) eyes open and (4) eyes closed (Figure 1A). The randomization of task order was conducted using a computer-generated randomization list created prior to data collection. In the parallel stance tasks, participants maintained the hip in a neutral position, with the feet pointed forward and kept together [17]. In the semi-tandem tasks, the dominant leg (control group) or the affected leg (knee osteoarthritis group) was positioned behind, with the hallux next to the contralateral heel [18]. For the tasks performed with eyes open, participants were instructed to fix their gaze on a target at eye level, positioned two meters away (Figure 1A). During all tasks, hands were placed on the waist [18]. Each task was performed twice, with each trial lasting 30 seconds. If a participant could not maintain the position, another attempt was made. A one-minute break was provided between tasks. The average of the two trials was considered for analysis. This protocol was chosen because it provides varying levels of postural difficulty through changes in base of support (feet together, semi-tandem) and sensory inputs (eyes open and closed). The reliability and sensitivity of this protocol were previously established by our research group [12].

Physical function tests

As recommended by the Osteoarthritis Research Society International [19], the TUG test and the 30-second chair stand test were used to assess physical function in participants with knee osteoarthritis. In the TUG test, participants were instructed to rise from a seated position, walk three meters, turn around, walk back to the chair, and sit down as quickly as possible. The path was linear and free of obstacles, and the total time to complete the task was recorded for further analysis. For the 30-second chair stand test, participants were seated in a standard chair with arms crossed over their chest and feet flat on the floor. On command, they were instructed to stand up and sit down as many times as possible within 30 seconds. The total number of completed stands was recorded for analysis.

Acceleration data analysis

Acceleration data were acquired from a smartphone (iPhone 7, software version 15.6.1; Apple Inc., Cupertino, CA, USA) equipped with a built-in 3D accelerometer, using the MATLAB Mobile app (free version; MathWorks, v. 8.9.1; MathWorks, Inc., Natick, MA, USA). The sampling frequency was set at 100 Hz, and the raw data were transmitted via Bluetooth to a personal computer and subsequently analyzed using a custom MATLAB script (MathWorks, v. R2020b).

The 3D acceleration signal (Figure 1B) was low-pass filtered at 5 Hz using a sixth-order Butterworth filter. The magnitude of acceleration was calculated using the Euclidean norm, combining the X, Y, and Z axes. To quantify body sway during each task, the root mean square (RMS) values were computed for the magnitude of acceleration, as well as for medio-lateral and antero-posterior acceleration. To minimize the impact of potential postural adjustments at the beginning and end of each task, the first and last five seconds of data were discarded, resulting in a 20-second window used for analysis.

Statistical analysis

As previous studies have demonstrated differences in body sway across postural conditions (base of support and visual input) [12,20], only between-group comparisons were performed. Given the non-normal distribution of the data, as determined by the Shapiro-Wilk test, the Mann-Whitney U-test was used to compare RMS values across postural tasks between the knee osteoarthritis and control groups.

The relationships between body sway variables and functional test scores in the knee osteoarthritis group were assessed using Pearson or Spearman correlation coefficients, depending on data normality (determined by the Shapiro-Wilk test). All statistical analyses were conducted using R (version 4.2.0; R Foundation for Statistical Computing, Vienna, Austria), with a significance level set at 5%.

## Results

Ability of body sway-related variables to differentiate groups

**Figure 2:**
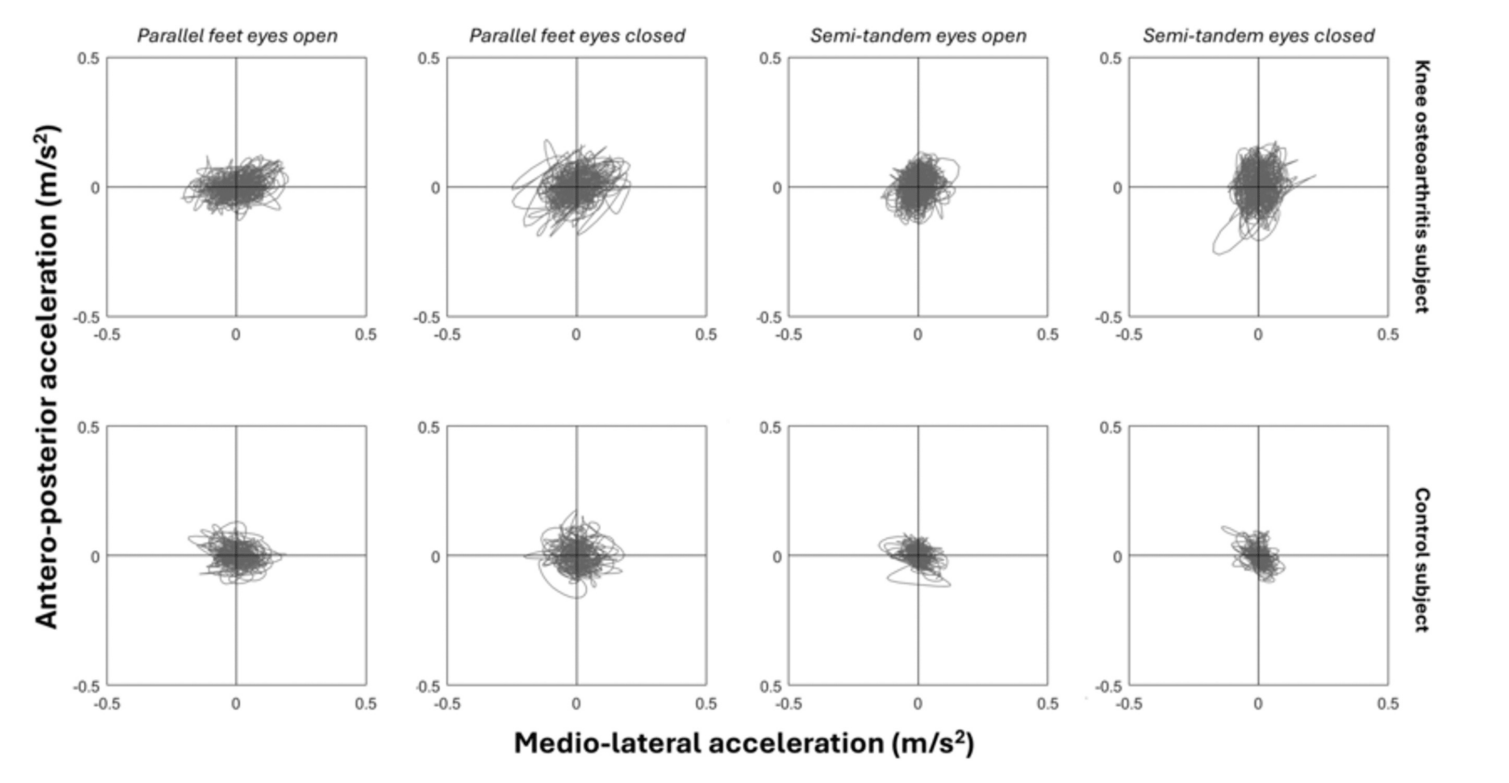
Representative cases Medio-lateral and antero-posterior acceleration during all postural balance tasks, performed by representative participants from the knee osteoarthritis and control groups.

To assess the ability of body sway-related variables collected via smartphone to differentiate between groups, we compared oscillation metrics between the knee osteoarthritis and control groups. Figure 2 illustrates the medio-lateral and antero-posterior acceleration (the vertical axis was omitted) during postural tasks performed by representative participants from each group. Visual analysis demonstrates increased body acceleration in both the medio-lateral and antero-posterior directions in the knee osteoarthritis group.

**Figure 3:**
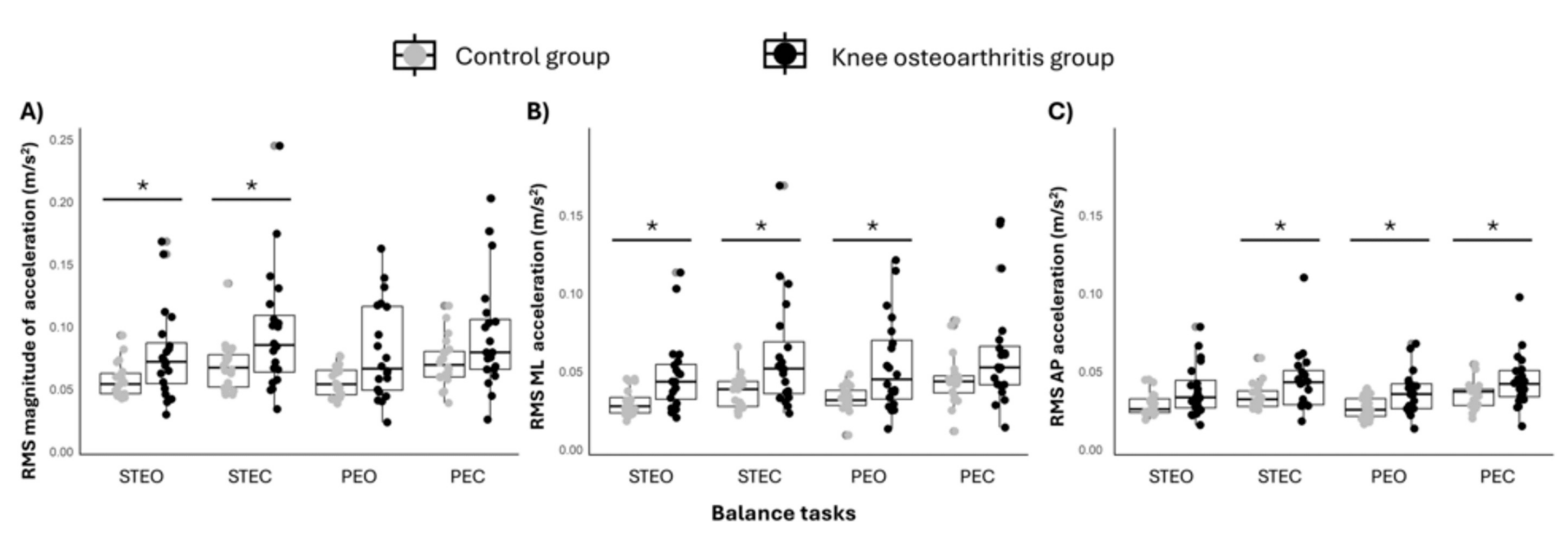
Comparison of body sway-related variables between groups The RMS magnitude of acceleration (panel A), RMS ML acceleration (panel B), and RMS AP acceleration (panel C) were compared across postural balance tasks: STEO, STEC, PEO, and PEC. Horizontal traces, boxes, and whiskers respectively denote the median value, interquartile interval, and distribution range. An asterisk indicates statistically significant differences between groups (p < 0.05). RMS, root mean square; ML, medio-lateral; AP, antero-posterior; STEO, semi-tandem with eyes open; STEC, semi-tandem with eyes closed; PEO, parallel feet with eyes open; PEC, parallel feet with eyes closed

The Mann-Whitney U-test revealed significant differences between the control and knee osteoarthritis groups across most tasks and parameters. In general, the knee osteoarthritis group exhibited significantly greater body acceleration compared to the control group (p < 0.04 for all cases; Figure 3). Exceptions included the parallel feet with eyes open and eyes closed for RMS magnitude of acceleration (Figure 3A); parallel feet with eyes closed for RMS medio-lateral acceleration (Figure 3B); and semi-tandem with eyes open for RMS antero-posterior acceleration (Figure 3C). All pairwise comparisons and respective effect sizes are presented in Table 1.

**Table 1:** Median and interquartile range of RMS magnitude and directional accelerations during balance tasks for control (CON) and knee osteoarthritis (KOA) groups AP, antero-posterior; ML, medio-lateral; PEC, parallel feet with eyes closed; PEO, parallel feet with eyes open; RMS, root mean square; STEC, semi-tandem with eyes closed; STEO, semi-tandem with eyes open

Balance task	Group (m/s^2^)		p-value	Effect-size r
	CON	KOA		
STEO				
RMS magnitude of acceleration	0.054 (0.016)	0.072 (0.032)	0.04	0.33
RMS ML acceleration	0.027 (0.009)	0.043 (0.022)	< 0.01	0.46
RMS AP acceleration	0.025 (0.008)	0.033 (0.017)	0.08	0.27
STEC				
RMS magnitude of acceleration	0.067 (0.025)	0.085 (0.045)	0.03	0.33
RMS ML acceleration	0.038 (0.016)	0.051 (0.033)	<0.01	0.42
RMS AP acceleration	0.032 (0.009)	0.043 (0.021)	0.03	0.33
PEO				
RMS magnitude of acceleration	0.054 (0.019)	0.066 (0.067)	0.10	0.26
RMS ML acceleration	0.031 (0.009)	0.044 (0.037)	0.01	0.38
RMS AP acceleration	0.025 (0.011)	0.035 (0.015)	0.01	0.40
PEC				
RMS magnitude of acceleration	0.069 (0.020)	0.079 (0.039)	0.13	0.24
RMS ML acceleration	0.044 (0.011)	0.053 (0.025)	0.11	0.25
RMS AP acceleration	0.037 (0.010)	0.042 (0.017)	0.04	0.32

Relationship between body sway and functional test performance

**Figure 4:**
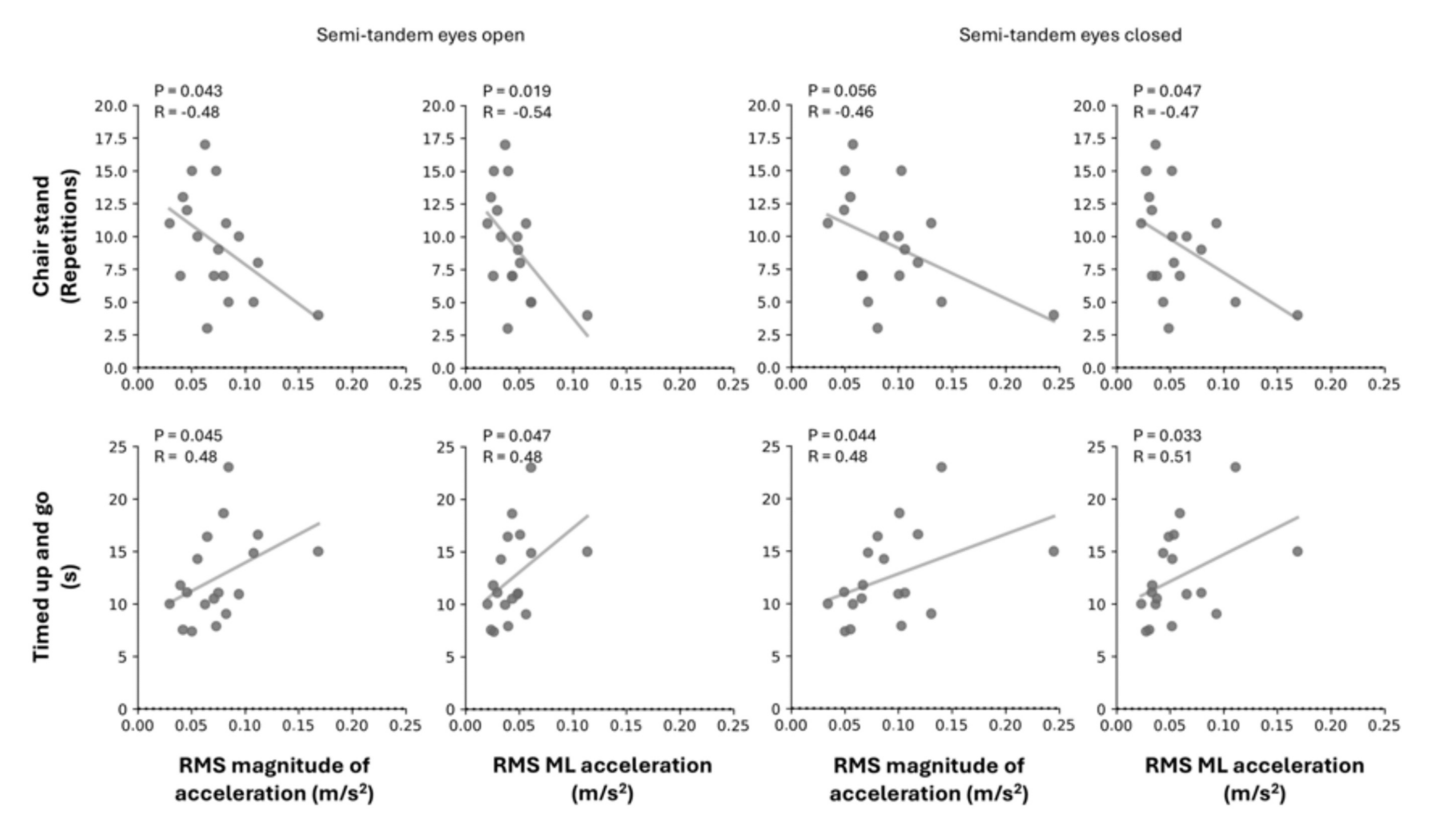
Correlation results Correlation between functional test performance and body sway variables, including RMS magnitude of acceleration and the RMS ML acceleration, was analyzed in the knee osteoarthritis group. RMA, root mean square; ML, medio-lateral

To investigate the relationship between static body sway and functional performance in knee osteoarthritis patients, we analyzed the correlations between body sway-related variables and the results of the TUG and 30-second chair stand tests. As two participants were unable to complete the functional tests, the analysis included 18 participants. The mean ± SD performance for the TUG was 13 ± 4 seconds, and for the 30-second chair stand test, it was 9 ± 4 repetitions. The strongest relationships between body sway variables and functional test performance are presented in Figure 4. A strong negative correlation (R = -0.73, p < 0.001) was observed between the TUG and the 30-second chair stand test. Additional correlation results are presented in Table 2.

**Table 2:** Correlation results between functional tests performance and body oscillation variables AP, antero-posterior; ML, medio-lateral; PEC, parallel feet with eyes closed; PEO, parallel feet with eyes open; STEC, semi-tandem with eyes closed; STEO, semi-tandem with eyes open; TUG, timed up and go

Balance task	TUG		30-second chair stand	
	R	p-value	R	p-value
STEO				
RMS magnitude of acceleration	0.48	0.045	-0.48	0.043
RMS ML acceleration	0.48	0.047	-0.54	0.019
RMS AP acceleration	0.34	0.164	-0.41	0.092
STEC				
RMS magnitude of acceleration	0.48	0.044	-0.46	0.056
RMS ML acceleration	0.51	0.033	-0.47	0.047
RMS AP acceleration	0.45	0.063	-0.37	0.126
PEO				
RMS magnitude of acceleration	0.24	0.328	-0.35	0.158
RMS ML acceleration	0.33	0.188	-0.44	0.066
RMS AP acceleration	0.28	0.252	-0.34	0.169
PEC				
RMS magnitude of acceleration	0.16	0.524	-0.38	0.117
RMS ML acceleration	0.23	0.348	-0.42	0.081
RMS AP acceleration	0.12	0.638	-0.39	0.105

## Discussion

The present study investigates the ability of smartphones to assess postural balance in patients with end-stage knee osteoarthritis. Our main findings demonstrate significantly higher body sway-related variables during quiet stance tests in the knee osteoarthritis group compared to healthy controls. Additionally, we explored the relationship between these body sway variables, the TUG test, and the 30-second chair stand test. Our results demonstrate that patients with reduced physical function exhibit increased body sway during semi-tandem tasks, especially in the medio-lateral direction.

Utility of the smartphone to assess postural control in patients with knee osteoarthritis

Knee osteoarthritis significantly impairs proprioception by damaging joint structures and surrounding muscles, leading to pain and edema that directly affect balance and, consequently, increase the risk of falls [21]. Indeed, a recent meta-analysis revealed that both symptomatic knee and hip osteoarthritis are associated with an increased risk of recurrent falls, while radiographic knee osteoarthritis is linked to an elevated risk of falls [22]. Given that falls and osteoarthritis represent major public health challenges, the widespread accessibility and cost-effectiveness of smartphones present a valuable opportunity for large-scale balance screening and monitoring. Therefore, utilizing smartphones in this context could enhance the management of fall risks and improve therapeutic interventions for individuals with knee osteoarthritis. 

Although smartphones have been used to assess postural control in several clinical populations [13,14], to the best of our knowledge, this is the first study investigating their use in patients with end-stage knee osteoarthritis. Previous studies using gold-standard equipment have demonstrated significant differences between individuals with knee osteoarthritis and healthy subjects in several postural sway metrics, as measured by a force platform [23] and a motion capture system [24]. Similarly, our results from smartphone acceleration data were also able to detect significant differences between knee osteoarthritis patients and healthy controls.

Our results demonstrated that body sway measurements derived from smartphone accelerometers are also sensitive in differentiating individuals with knee osteoarthritis from healthy controls, especially when acceleration in the medio-lateral direction during semi-tandem tasks is analyzed. Although the knee osteoarthritis group showed higher body acceleration values than the control group in most tasks performed and for the three body sway metrics used (Figure 3), the largest effect sizes were observed in the medio-lateral acceleration during semi-tandem tasks with eyes open and closed (Table 1). This pronounced sensitivity in medio-lateral acceleration during semi-tandem tasks can be attributed to the narrowed stance adopted, which promotes changes in the postural coordination of lower limb joints [25,26] and an increased reliance on ankle-surrounding muscles, such as the tibialis anterior [20]. Another relevant factor is the increased effort required from the limb recommended for total knee arthroplasty, as this limb is positioned posteriorly and receives more weight discharge. In contrast, the parallel feet task allows for greater compensation by the contralateral limb. Therefore, these findings indicate that medio-lateral body sway measurements during semi-tandem tasks are particularly appropriate for identifying and monitoring postural control strategies in individuals with unilateral end-stage knee osteoarthritis.

Smartphone-derived body sway measurements associated with functional test performance in knee osteoarthritis

We observed significant correlations between smartphone-derived body sway measurements and the performance of functional tests in patients with end-stage knee osteoarthritis (Figure 4). Increased medio-lateral acceleration, particularly during semi-tandem tasks, was associated with poorer performance on the TUG test. This finding may be explained by the fact that patients with knee osteoarthritis often exhibit weakness in muscles that stabilize the medio-lateral plane, such as the gluteus medius and gluteus maximus [27]. These muscles are crucial for single-leg balance in the medio-lateral plane - a key component of walking [28] - and the strongest relationships between TUG performance and acceleration in the medio-lateral direction (Table 2) further support this. Moreover, increased body sway can be associated with deficits in lower limb strength and endurance, commonly observed in patients with knee osteoarthritis [29], as evidenced by significant correlations between smartphone-derived body sway measurements and the 30-second chair stand test.

The significant correlation between smartphone-derived sway measurements and functional performance underscores the potential use of this technology in clinical practice. Functional tests are recommended because they reflect the physical abilities relevant to individuals diagnosed with knee osteoarthritis [19]. However, in severe cases, pain and stiffness often limit the ability to perform even simple tasks, such as walking and sitting. Our findings highlight the potential of smartphone accelerometry not only to assess postural balance but also to infer functional impairments in patients with end-stage knee osteoarthritis - particularly when patients might avoid more strenuous tests due to joint discomfort. Additionally, these measures could help clinicians identify individuals at greater risk of functional decline and guide personalized rehabilitation interventions aimed at improving balance and physical function.

Limitations and future perspectives

The main limitation of this study is that the control group was not age-matched with the knee osteoarthritis group, which could introduce age-related variability in balance performance. However, previous studies have already demonstrated significant differences in postural sway between age-matched groups [23,30], suggesting that - even without age matching - the findings of increased body sway in the knee osteoarthritis group may still be valid. Additionally, another limitation is the severity of knee osteoarthritis, as we assessed patients in the end stage of the condition. This focus may limit the extrapolation of findings to individuals with earlier stages of knee osteoarthritis, who may exhibit different balance characteristics and functional impairments.

Further investigations should focus on integrating smartphone-based assessments of postural balance into clinical practice, enhancing accessibility and enabling routine monitoring of knee osteoarthritis patients on a large scale. Furthermore, developing user-friendly mobile applications for balance assessment could empower patients to self-monitor and manage their condition more effectively.

## Conclusions

In conclusion, our study demonstrates that individuals with knee osteoarthritis exhibit significantly greater body sway during quiet stance tests compared to healthy controls. Additionally, our results reveal a relationship between smartphone-derived body sway measures and performance in physical tests. Increased acceleration - especially in the medio-lateral direction during semi-tandem tasks - was associated with poorer functional outcomes. This finding suggests that smartphone technology can effectively identify balance deficits that correlate with reduced physical function in patients with end-stage knee osteoarthritis. Overall, this study represents a significant advancement in integrating smartphone technology into clinical practice for assessing and managing postural control, opening new possibilities for future research and practical applications in patient care.
